# Dopamine D3 Receptor Plasticity in Parkinson’s Disease and L-DOPA-Induced Dyskinesia

**DOI:** 10.3390/biomedicines9030314

**Published:** 2021-03-19

**Authors:** Kathryn Lanza, Christopher Bishop

**Affiliations:** 1 Department of Physiology, Northwestern University, Chicago, IL 60201, USA; kathryn.lanza@northwestern.edu; 2 Department of Psychology, Binghamton University, Binghamton, NY 13902, USA

**Keywords:** dopamine D3 receptor, dopamine D1 receptor, D1R–D3R, Parkinson’s Disease, L-DOPA-induced dyskinesia, striatum

## Abstract

Parkinson’s Disease (PD) is characterized by primary and secondary plasticity that occurs in response to progressive degeneration and long-term L-DOPA treatment. Some of this plasticity contributes to the detrimental side effects associated with chronic L-DOPA treatment, namely L-DOPA-induced dyskinesia (LID). The dopamine D3 receptor (D3R) has emerged as a promising target in LID management as it is upregulated in LID. This upregulation occurs primarily in the D1-receptor-bearing (D1R) cells of the striatum, which have been repeatedly implicated in LID manifestation. D3R undergoes dynamic changes both in PD and in LID, making it difficult to delineate D3R’s specific contributions, but recent genetic and pharmacologic tools have helped to clarify its role in LID. The following review will discuss these changes, recent advances to better clarify D3R in both PD and LID and potential steps for translating these findings.

## 1. Introduction

Parkinson’s Disease (PD) is the second-most-common neurodegenerative disorder and is primarily characterized by the death of midbrain dopaminergic neurons in the substantia nigra pars compacta. The loss of dopaminergic inputs to the striatum results in the manifestation of PD’s cardinal motor symptoms: bradykinesia, akinesia, postural instability and rigidity [1]. However, behavioral manifestations of PD do not appear until significant cell death has already occurred [2]. This is due to the fact that nuclei within the basal ganglia undergo significant changes to compensate for the progressive loss of dopaminergic cells. Some of this compensation is clearly evidenced in morphological studies that show enlarged remaining nigrostriatal terminals, innervation of projections from non-dopaminergic areas and post-synaptic dendritic sprouting [3]. Additionally, changes to dopamine receptor localization, signaling and function have all been demonstrated in PD.

Over 60 years ago, L-DOPA was discovered as an effective treatment to replenish the loss of endogenous dopamine [4]. Although L-DOPA remains the gold-standard pharmacotherapy and initially provides motor benefit, chronic treatment inevitably results in L-DOPA-induced dyskinesia (LID) in up to 95% of patients after 15 years of treatment [5,6]. Given that L-DOPA therapy is normally started in mid-stage PD, the previously described neuroplasticity that compensates in post-symptomatic PD can indicate the individual for LID. As PD progresses and LID manifests, dopamine receptors exhibit dynamic plasticity that differentially participates in PD/LID. The following review will track the plasticity of dopamine receptors, namely the dopamine D3 receptor (D3R), throughout PD and LID. In the past decade, multiple pharmacologic (Table 1) and non-pharmacologic (Table 2) strategies have revealed the therapeutic potential of targeting D3R.

## 2. The D3 Receptor

The dopamine D3 receptor (D3R) was first molecularly cloned and characterized in 1990 [7,8]. Since its discovery, D3R has emerged as a promising yet enigmatic target for treatment in PD, substance abuse and schizophrenia [9,10]. D3R belongs to the dopamine D2-like (D2R, D3R, D4R) family of G-protein-coupled receptors (GPCRs), which, prototypically, are Gi-coupled and act by inhibiting adenylate cyclase (AC) signaling and downstream effectors [11]. This is in opposition to the D1-like (D1R, D5R) family of receptors, which are Gs/q/olf-coupled and positively regulate downstream signaling. D3R shares 75–80% transmembrane domain homology with D2R [11]. However, the structural differences that do exist, mainly in loop regions, between D2R and D3R result in profound functional outcomes. The crystallization of D3R in 2010 revealed the presence of an allosteric extracellular binding pocket in D3R that likely contributes to the widely variable responses of D3R-targeting compounds [12]. Additionally, whereas D2R rapidly internalizes upon stimulation, D3R displays little agonist-induced internalization. This is likely due to differences in intracellular loops which, when swapped, reversed the internalization profile of D2R and D3R.

Notably, D3R possesses the highest affinity for dopamine and potently interacts with many putative D2R-like agonists [7]. Therefore, minor changes to D3R expression may significantly modify dopaminergic signaling. Neural D3R expression is more restricted than D1R and D2R, which display diffuse expression throughout functionally heterogenous structures of the brain. D3R is robustly expressed in the islands of Calleja, the ventromedial shell of the nucleus accumbens, the substantia nigra, olfactory tubercle and in some areas of the cerebellum [7,13,14]. A population of D3R also exists in the pyramidal cells of the prefrontal cortex, where they regulate cell excitability via a subtype of calcium channels [15]. D3R has also been pharmacologically interrogated in the rat hippocampus, where D3R is post-synaptically situated [16].

## 3. Dopamine D3 Receptor (D3R) in the Parkinsonian Brain

### 3.1. Dopamine D1 Receptor (D1R) and Dopamine D3 Receptor (D3R) Expression Following Denervation

**Figure 1:**
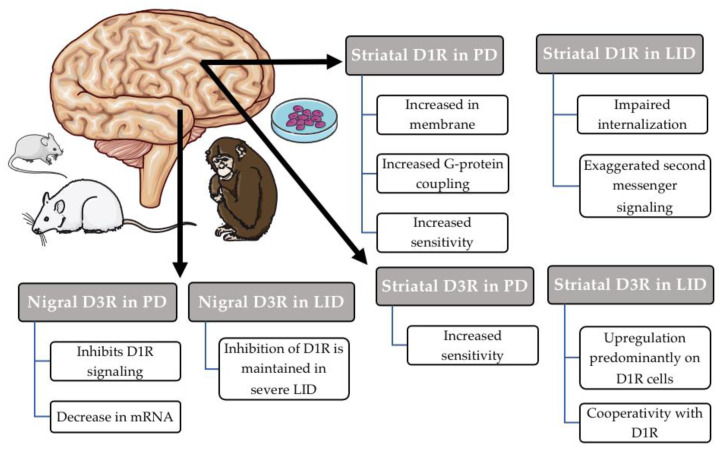
Summary of major changes associated with dopamine D1/D3 receptors in the striatum and nigra. Studies in cells, mice, rats, non-human primates and humans have demonstrated dynamic changes to dopamine receptors D1 (D1R) and D3 (D3R) across Parkinson’s Disease (PD) and L-DOPA-induced dyskinesia (LID). In the denervated state, D1R are sensitized. This is partially achieved by increases in membrane-bound G-protein-coupled D1R. In L-DOPA-induced dyskinesia (LID), D1R are unable to internalize and effectively terminate signaling, leading to further supersensitization and second messenger signaling. Some evidence suggests that striatal D3R are also supersensitive in PD but are expressed at almost undetectable levels. In LID, D3R are upregulated predominantly on D1R cells, where they display cooperativity with D1R at the level of downstream signaling. In contrast, in striatonigral terminals, D3R inhibits D1R signaling in the denervated state. This inhibitory property is maintained in severely (but not mildly) dyskinetic subjects. Image credit: Servier medical art (http://smart.servier.com/; Access date: 15 March 2021).

Parkinson’s Disease (PD) is characterized by the progressive and irreversible loss of midbrain dopaminergic cells. The striatum normally receives dense dopaminergic input from these cells and undergoes significant changes to compensate for the loss of dopamine. Changes to dopamine receptor expression, largely on medium spiny neurons (MSNs), are one way in which this compensation manifests. This is clearly evident in D1R subcellular localization and function. D1R shares 75–80% transmembrane domain homology with the other D1-like receptor, D5R [17]. However, in terms of relative expression in the brain, D1R dominates. It displays high levels of expression in the caudate–putamen, nucleus accumbens, substantia nigra, olfactory bulb, amygdala and frontal cortex. In the hippocampus, cerebellum, thalamus and hypothalamic areas, D1R is also expressed but at lower levels [11,17]. D1R can localize on MSNs both post-synaptically in opposition to incoming DA afferents and pre-synaptically to modulate γ-Aminobutyric acid (GABA) release in output nuclei such as the substantia nigra pars reticulata [18]. It does not appear that changes to overt D1R expression significantly contribute to D1R receptor sensitivity, although this remains a matter of debate. Rather, changes to D1R localization within the cell may be more important (Figure 1). In 2007, Guigoni and colleagues found increased D1R immunoreactivity in the striatum of 1-methyl-4-phenyl-1,2,3,6-tetrahydropyridine (MPTP)-treated primates specifically in perimembranous regions, indicating increased D1R recruitment to the plasma membrane [19]. A nearly identical result was also found in hemi-parkinsonian rats [20]. The consequences of increased expression of D1R at the cell surface have implications in LID. Interestingly, in a genetic model of PD, mice expressing the disease-associated mutant G2019S LRRK2 also displayed impaired internalization of D1R [21]. Alterations in D1R trafficking may be a hallmark of both idiopathic and sporadic forms of PD.

Other changes occur to D1R in the DA-denervated brain related to G-protein coupling and downstream effectors. As previously described, D1R canonically couples with Gαs. However, D1R has been observed to also couple with other stimulatory G-proteins such as Gα_olf_, particularly in the rodent striatum. In response to 6-OHDA lesion, Gα_olf_ expression is increased in the striatum, as is DA-dependent AC activity [22,23]. Though others have found that gross Gα_olf_ expression is not changed [24], D1R coupling with Gα_olf_ is enhanced according to co-immunoprecipitation. Therefore, this shift has been postulated to play a role in the supersensitivity of D1R before L-DOPA initiation begins. Notably, Gα_olf_ levels were also increased in postmortem PD brains [23]. In the same vein, increased levels of Gα_olf_-dependent AC type 5 (AC5) have also been observed in both the striatum and substantia nigra pars reticulata in hemi-parkinsonian rats, suggesting enhanced downstream signaling in addition to G-protein coupling [25]. Therefore, converging evidence suggests that a shift to a more sensitive state related due to cellular localization, G-protein coupling and downstream signaling contributes to D1 sensitivity following DA cell loss (Figure 1). In the past few years, the role of other DA receptors, namely D3R, has become increasingly clear.

As mentioned, D3R expression in the rodent is considerably more restricted than expression of D1R and D2R. Interestingly, despite the fact that the ventral striatum is less affected in PD modeling, D3R mRNA and binding is reduced in the nucleus accumbens following a 6-OHDA lesion in rats or MPTP treatment in monkeys [26]. In tandem, D3R mRNA in the substantia nigra reticulata is decreased [7]. The consequences of these changes in expression are not well understood. In the striatum, where D3R expression is considerably lower, subtle changes in expression following dopamine depletion might be difficult to detect. In rats, striatal D3R binding does not change in response to 6-OHDA lesion [27]. In MPTP-treated monkeys, decreases in D3R binding have been observed in the caudate but not in the putamen [28,29,30]. In humans, in vivo imaging of D2-like receptors with [^11^C] Raclopride demonstrated an increase in D2-like receptors in early PD within the putamen [31]. In contrast, D3R expression was not changed in postmortem samples from PD patients [32]. In human studies, these observational differences in D3R expression may be because D3R undergoes distinct changes during disease progression and then again during L-DOPA treatment. Discrepancies in these observations might also be related to the species-specific differences in D3R expression compounded with a lack of pharmacological probe specificity. In general, it seems that in species where D3R is normally not expressed at detectable levels in the striatum (e.g., rodents), DA denervation does not significantly change D3R expression. However, in animals where D3R is lowly expressed (e.g., non-human primates and humans), D3R expression undergoes modest changes in expression that depend on disease duration. The use of D3R expression as a biomarker in prodromal/preclinical PD is extensively discussed in [33].

### 3.2. Dopamine D3 Receptor (D3R) Signaling Changes Following Denervation

**Figure 2:**
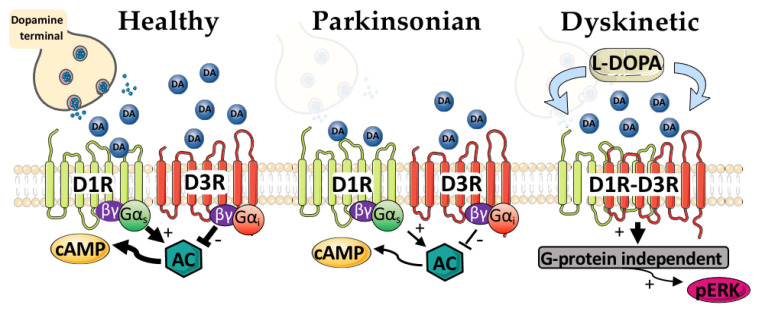
Theoretical signaling pathways of the dopamine D1/D3 receptors in the healthy, Parkinsonian or dyskinetic striatum. In the intact brain, dopamine (DA) is released from striatonigral terminals and interacts with post-synaptic D1 receptors (D1R; low DA affinity but high expression) and dopamine D3 receptors (D3R; high DA affinity but low expression) which couple with canonical G-proteins to increase or decrease G-protein signaling, respectively. Each of these receptor properties is theoretically maintained in PD, with reductions in DA levels due to the retraction of striatonigral terminals. D1R is sensitized due to increased presence at the membrane. In the dyskinetic state, DA is exogenously provided via L-DOPA. D3R is upregulated and interacts with D1R, potentially in the form of a heteromer. D1R–D3R cooperate to drive downstream signaling, such as phosphorylation of ERK (pERK). This signaling might be G-protein-independent. cAMP: cyclic AMP; AC: adenylyl cyclase. Image credit: Servier medical art (http://smart.servier.com/; Access date: 15 March 2021).

Although overt changes to D3R expression in PD are still not completely clear, parameters related to D3R signaling do change in response to dopamine cell loss (Figure 1 and Figure 2). Following 6-OHDA striatonigral lesions, the D3R agonist 7-OH-DPAT displays enhanced potency in the striatum [34]. Moreover, electrophysiological and behavioral studies suggest that D3R activity may be enhanced, displaying a supersensitive profile similar to D1R [35,36]. The mechanisms for D3R supersensitization are not entirely clear, but one of these may relate to D3R interactions with its truncated receptor splice variant D3nf. One of the purposes of truncated receptors is to modulate the activity of full-length receptors at the cell surface. Therefore, when it is colocalized and interacting with D3R, D3nf decreases the capacity of D3R to interact with ligands. Notably, this does not correspond with decreased membrane localization of D3R, explaining why differences in ligand binding in PD may or may not be observed [37]. Moreover, D3R/D3Rnf ratios are sensitive to hyper- and hypodopaminergic states [38]. Neurotoxic 6-OHDA lesion significantly reduced D3nf protein in the striatum of rats [35]. As a result, the internal regulatory mechanisms of D3R via the production of D3nf may be compromised in the DA-denervated state and contribute to D3R supersensitivity [39].

D3R changes in response to denervation have also been reported downstream from the striatum in striatonigral terminals (Figure 1). In the intact brain, within these terminals, D3R interacts with D1R to potentiate GABA release [18,40]. Although this is atypical signaling for D3R in terms of its canonical G-protein, others have reported similar effects when D1R and D3R colocalize [41,42]. Importantly, within the SNr, the ability of D3R to potentiate D1R signaling is dynamic and depends on levels of cytoplasmic Ca^2+^. When calcium/calmodulin-dependent protein kinase II (CaMKII) is activated by Ca^2+^, CaMKII phosphorylates the third intracellular loop of D3R. In this phosphorylated state, D3R is no longer able to potentiate D1R signaling [40,43]. However, in the 6-OHDA lesioned rat, this property of D3R seems to malfunction [44]. Regardless of CaMKII levels, D3R is unable to potentiate D1R levels and D3R actively suppresses D1R signaling following D1R stimulation, suggesting that D3R switches from typical (potentiating) to atypical (inhibiting) signaling. Interestingly, this switch is not applied ubiquitously across all D3R in striatonigral terminals, as both typical and atypical signaling is observed [45]. Collectively, these data highlight the fact that dopamine depletion differentially modifies D3R’s actions in the basal ganglia.

## 4. Dopamine D3 Receptor (D3R) in the Dyskinetic Brain

### 4.1. Dopamine D3 Receptor (D3R) Changes to Expression in LID

Dopamine replacement therapy with L-DOPA remains the gold-standard pharmacotherapy to mitigate the symptoms of PD. However, with chronic use, LID develops in the majority of patients. Previous plasticity of dopamine receptors, such as the increased synaptic localization of D1R or supersensitization of D3R, that helped to compensate for dopamine loss likely contributes to LID manifestation. In the striatum, D3R changes both in expression and function. As early as 1997, Bordet and colleagues noted that the ectopic expression of D3R in the striatum occurs as a result of L-DOPA administration and actively participates in behavioral sensitization [46]. Since then, this phenomenon has been replicated across multiple other laboratories and models of LID. In 6-OHDA-lesioned rats and mice [27,47,48], MPTP-treated mice [49] and MPTP-treated monkeys [27,30,50], dyskinetic subjects display enhanced D3R expression. In monkeys, L-DOPA normalizes the MPTP-induced decrease in D3R in the caudate, but levels are increased beyond controls in animals with LID [29]. Furthermore, putaminal D3R levels and LID expression are positively correlated, a trend that has not been observed for any other DA receptor subtype. This upregulation is largely, if not exclusively, post-synaptic, considering that most DA terminals are degraded at this late stage [30]. Although experimental overexpression of D3R alone is enough to produce some stereotyped behaviors [51], it appears that endogenous upregulation depends on both DA loss and subsequent L-DOPA treatment, as neither denervation nor L-DOPA alone produces significant upregulation [49]. In rats rendered dyskinetic with chronic L-DOPA, D3R agonism results in profound dyskinesia in a dose-dependent manner [52]. Upregulation of D3R has also been reported in striatal output nuclei within the globus pallidus interna in both monkeys [28] and humans with a history of L-DOPA treatment [53,54].

D3R normally displays low expression in the dorsal striatum, which makes upregulation of D3R notable in and of itself. Blocking D3R upregulation via intrastriatal infusion of oligonucleotide antisense to dopamine D3R mRNA attenuated the development of LID in the 6-OHDA rodent model [55]. Though D3R is traditionally considered a member of the D2-like family, upregulation of D3R predominantly occurs on D1R-bearing direct-pathway MSNs [27,46,47,48]. Although D3R might upregulate, to a lesser degree, on D2R-bearing MSNs, the specific upregulation on direct-pathway MSNs likely plays a causal role in LID manifestation. In 2017, Solis et al. found that global knockout of D3R not only attenuates LID but also reduces direct-pathway-associated markers of LID (Table 2). The same study used a combination of genetic and pharmacological approaches to better clarify the relationship between D1R and D3R. Heterozygous D1± mice that were administered a D3R antagonist in conjunction with L-DOPA displayed reduced LID compared to both WT and D1± mice receiving L-DOPA alone (Table 1 and Table 2). Furthermore, this group had lower expression of FosB and acetylation of histone 3, which they previously identified to occur primarily in D1R+ striatal cells. It appears that D1R and D3R are undeniably interacting within the same cell in LID. Within these cells, some data suggest a physical interaction of D1R–D3R in the form of a heteromer. In transfected mammalian systems and striatal membrane preparations, D1R and D3R form heteromers [41,42]. One consequence of this interaction is that D3R effectively makes D1R resistant to individual agonist-induced internalization, making D1R “locked” in the membrane [41]. In 2014, Guitart and colleagues expanded these findings to show that, in vitro, D1R and D3R form higher-order heterodimers in the form of two homodimeric complexes [56]. Whether or not this complex exists in vivo remains to be determined, but, minimally, in both the rat and monkey striatum, D1R–D3R heteromers are detectable in dyskinetic subjects [27]. Very recently, researchers found that in human brain samples, D1R–D3R densities better predicted disease progression and treatment than either receptor alone [57]. We recently directly tested the dyskinesiogenic effect of D3R by injecting a D3R miRNA into the striatum of D1-Cre rats prior to chronic L-DOPA treatment. As summarized in Table 2, we showed that region- (striatal) and cell- (D1R- cells in the striatum) specific knockdown of D3R results in attenuated LID development without compromising L-DOPA’s therapeutic benefits [58]. Whether or not this resulted in a reduction in D1R–D3R heteromers is not known. However, anti-dyskinetic strategies reduce D1R–D3R heteromers, suggesting that these interactions are indeed specific to LID [59].

The mechanism of D3R upregulation is not fully understood. However, D3R expression can be bidirectionally modified with D1R agonism/antagonism, suggesting a role of D1R stimulation in recruitment of D3R [46,47]. In support of this, we previously demonstrated that D1R and D3R agonism results in cross-sensitization. Prior sub-chronic exposure to either a D1R or D3R agonist results in a sensitized dyskinetic response when the other agonist is acutely administered [68]. There are some data linking D1R stimulation and D3R expression with elevated expression of striatal brain-derived neurotrophic factor (BDNF), an important factor in cellular proliferation, differentiation and survival [69]. In heterozygous BDNF mice, D3R mRNA is lower than in wildtype controls within the striatum. Exogenous intrastriatal administration of BDNF partially restores D3R mRNA [70]. In the context of PD/LID, Guillan and colleagues found that BDNF antagonism during L-DOPA treatment blocks D3R upregulation and behavioral sensitization in 6-OHDA-lesioned rats [71]. In this work, these authors suggest that BDNF originating from hyperactive corticostriatal projections works in conjunction with D1R agonism to recruit D3R to the striatum. Although overexpression of striatal BDNF alone increases D3R receptor expression, this also exacerbates both LID and D1R-agonist induced dyskinesia [59,72]. BDNF overexpression also increased the expression of D1R–D3R heteromers [73]. There is clearly a link between D1R stimulation, BDNF expression and induction of D3R. Whether or not there is an opportunity to leverage this therapeutically remains unclear. This effect is also not entirely consistent across other models of PD/LID. In MPTP-treated monkeys, levels of BDNF are not related to LID expression [74]. Although others have found that levels of BDNF are at least correlated with LID [75], the direct role of BDNF in D3R expression remains somewhat speculative.

### 4.2. Dopamine D3 Receptor (D3R) Changes to Signaling in LID

By itself, D3R can signal both G-protein-dependently and independently. In MPTP-treated mice rendered dyskinetic, a low dose of a D3R agonist (PD128907; 0.05 mg/kg) results in blunted MAPK signaling [49]. In contrast, we found that a low but dyskinesiogenic dose (0.1 mg/kg) of the same D3R agonist does not modify striatal pERK1/2 signaling in 6-OHDA-lesioned dyskinetic rats [52]. This discrepancy could be related to the PD model, dose of drug or timing of tissue collection. The employment of recently available PKA and ERK1/2 sensors will be important in delineating the timeline of intracellular signaling, which can fluctuate significantly during the duration of treatment [76]. Downstream in striatonigral projections, D3R agonism alone does not modify cAMP accumulation in synaptosomal preps but D1R–D3R co-stimulation does modify both GABA release and cAMP accumulation. In the denervated state, this potentiation is lost and D3R becomes antagonistic to D1R signaling [44]. The antagonistic relationship between D1R and D3R was only eliminated in mildly dyskinetic subjects, whereas this relationship remained in severely dyskinetic subjects.

In the striatum, a different pattern emerges in LID between D1R and D3R. This relationship is cooperative, rather than antagonistic. D3R antagonism during L-DOPA treatment in heterozygous D1± reduces direct-pathway markers associated with cellular activation, suggesting a cooperative, rather than antagonistic, interaction between the two receptors in terms of signaling [48]. In in vitro preparations, D1R–D3R complexes signal through G-protein-independent signaling cascades, where D1R–D3R maintain a canonical antagonistic relationship at the level of cAMP but synergistically cooperate to drive phosphorylation of ERK [56]. A similar, G-protein-independent, functional selectivity was also found in the nucleus accumbens, where D1R–D3R basally interact [77]. It remains to be determined if a similar pattern of signaling occurs in vivo in the dorsal striatum, where D1R–D3R interactions become ectopically expressed (Figure 2). We previously demonstrated that systemic coadministration of D1R and D3R agonists results in synergistic increases in both dyskinesia and striatal expression of pERK1/2 [52], supporting previous research demonstrating the site-specific cooperativity of D1R–D3R in downstream signaling [41,77].

## 5. Targeting D3R in LID

Given the abundance of data suggesting a dyskinesiogenic role of D3R, it is not surprising that this has emerged as an intriguing pharmacological target to manage LID (Table 1). Given that D3R potentiates D1R activity, strategies to normalize D3R function (either via partial agonism or antagonism) could interfere with this reciprocal relationship. In line with this, Bézard and colleagues administered BP 897 to MPTP-intoxicated macaques rendered dyskinetic by chronic L-DOPA. BP 897 is a mixed D3R partial agonist and antagonist (depending on dose). When administered with L-DOPA, BP 897 attenuated LID by 66% without affecting L-DOPA’s antiparkinsonian effects [28]. However, BP 897 did interfere with L-DOPA’s efficacy in squirrel monkeys, where the dosage resulted in higher plasma levels that might modify BP 897’s selectivity for D3R alone [60], suggesting a narrow therapeutic window for this compound. Paradoxically, both a highly selective (ST 198) and less specific (nafadotride) D3R antagonist also interfered with L-DOPA efficacy in the MPTP macaque model [28]. In marmosets, twice daily coadministration of S33084, a selective competitive D3R antagonist, resulted in attenuated LID development over the 30-day treatment period compared to L-DOPA alone subjects. This did not come at the expense of L-DOPA’s antiparkinsonian actions. Following a 2-week washout period, previously S33084-treated subjects still displayed lower LID in response to a L-DOPA challenge, but this effect was lost in 50% of subjects at the next 2-week timepoint [63]. The same study also tested the efficacy of S33084 to alleviate L-DOPA sensitization (as assayed by rotational behavior) in the 6-OHDA rat model and found that D3R antagonism effectively reduced L-DOPA sensitization development, but not expression. Similarly, another group found that S33084 did not affect established LID in MPTP-intoxicated marmosets, although it did improve the antiparkinsonian actions of L-DOPA and ropinirole [78]. Data from 6-OHDA-lesioned rats support these findings, with S33084 not improving LID development or expression but potentially improving Parkinsonian disability, either as a monotherapy or adjunctive treatment to L-DOPA [64]. Similarly, the D3R antagonist GR103691 had no effect on LID when administered as a co-treatment to L-DOPA [66].

However, some D3R antagonists are more promising in ameliorating LID, even if the mechanisms by which they convey their effects remain elusive. Administration of the D3R antagonist PG01037 both before and after L-DOPA administration significantly reduced LID expression in dyskinetic rats, with the 15 min post-L-DOPA regimen being the most effective [61]. PD1037 also interfered with apomorphine-, but not D1R agonist-, evoked dyskinesia. Another group confirmed the anti-dyskinetic effects of PG01037 when administered 15 min post-L-DOPA [48]. In 2016, Sebasianutto and colleagues demonstrated that even lower doses of PG01037 can reduce AIM expression without impacting other locomotor measures [62]. The structurally similar partial D3R agonist PG01042 also reduced LID when coadministered with L-DOPA, mimicking the previously observed effects of D3R partial agonism of BP 897 [28,65]. Similarly, the D3R partial agonist SK609 improved LID, L-DOPA’s therapeutic effect and even cognitive performance in a PD model [67,79]. To our knowledge, there is no evidence that an anti-dyskinetic D3R antagonist might worsen Parkinsonian symptoms.

Clearly, D3R-targeting compounds are highly variable (Table 1). There are several reasons that these contradictions in the literature may exist. The simplest explanation is that of specificity. The aforementioned similarity in D2R and D3R helps to explain many of these cases, in that “D3R” tools are not entirely selective for D3R. However, inconsistencies persist even when using highly selective D3R pharmacological probes. As discussed, D3R signaling is extremely diverse and D3R-specific ligands likely engage this diversity, biasing D3R signaling towards one or more intracellular pathway. Biased agonism that results in functional GPCR selectivity is being described more frequently, both behaviorally and cellularly, particularly with DA receptors [80]. Additionally, allosteric modulation because of heteromerization is a common outcome across several GPCR heteromers [80]. Given that D1R–D3R heteromers might signal G-protein-independently to synergistically drive downstream signaling [52,56,77], engaging G-protein-dependent signaling of D3R (either via partial agonism or antagonism) or targeting D1R–D3R cooperativity might be the key to unlocking D3R’s therapeutic potential [9]. Furthermore, region-specific understanding of D3R is warranted. D3R may play an antagonistic [44] or cooperative [48,52] role in D1R depending on where it is localized within the basal ganglia. D3R also forms heteromers with other receptors beyond D1R, which further contributes to its functional selectivity, signaling diversity and DA processing [81,82,83,84]. For many years, a lack of ligand specificity hindered the ability to rigorously test D3R’s role in the brain and behavior. Now, more D3R tools are available and being used in vivo to accomplish this goal [9,85].

## 6. Conclusions and Future Directions

Since its discovery in 1990, D3R has been implicated as a key player in many disorders characterized by dopamine dysfunction [7,8]. Indeed, as early as 1997, Bordet and colleagues linked D3R upregulation to behavioral sensitization to L-DOPA [46]. Shortly after, the same group connected D1R stimulation to D3R upregulation in a model of PD [47]. D1R–D3R interactions have been thoroughly characterized in vitro [41,42,56] but many questions about their in vivo significance still remain. Cell-specific targeting of D3R has revealed a dyskinesiogenic role of D3R, but how this might be leveraged therapeutically remains an open question [58]. Despite early studies suggesting that D3R has limited therapeutic potential [28,60,64,78], the generation of new compounds that take into account the unique signaling properties of D3R has reinvigorated the field. D3R is a promising target once again [9,67]. In fact, the targeting of D3R to alleviate LID is already being translated clinically. The D3R antagonist IRL790 (Mesdopetam) is currently undergoing clinical trials for LID management in PD patients. Early phases suggest that IRL790 is well-tolerated and effective in reducing LID [86,87] and phase IIB trials (NCT04435431) are currently underway. As more information is gathered regarding the structural relationship between D3R and its binding/signaling partners, the number of highly specific D3R compounds is only expected to rise [12,88]. These developments have the potential to impact both LID management and a number of disorders marked by aberrant D3R activity.

## Figures and Tables

**Table 1:** Pharmacologic strategies to target D3R.

Compound (Action)	Model	Effect on LID	L-DOPA Efficacy	Ref.
ST 198 (antagonist)	MPTP macaque	↓ expression	↓	[28]
BP 897 (partial agonist)	MPTP macaque	↓ expression	=	[28]
	MPTP squirrel monkey	↓ expression	↓	[60]
PG01037 (antagonist)	Striatal 6-OHDA mice	↓ expression	=	[48]
	Striatal 6-OHDA mice	↓ development	=	[48]
	MFB 6-OHDA rats	↓ expression	=	[61]
	MFB 6-OHDA mice	↓ expression	=	[62]
S33084 (antagonist)	MPTP marmoset	↓ development	=	[63]
	MFB 6-OHDA rats	↓ development of sensitization	?	[63]
	MFB 6-OHDA rats	- expression of sensitization	?	[63]
	MFB 6-OHDA rats	- development	↑	[64]
	MFB 6-OHDA rats	- expression	↑	[64]
	MPTP marmoset	- expression	↑	[65]
GR103691 (antagonist)	MFB 6-OHDA rats	- expression	=	[66]
PG01042 (agonist)	MFB 6-OHDA rats	↓ expression	=	[65]
SK609 (agonist)	MFB 6-OHDA rats	↓ expression	↑	[67]

Summary of pharmacologic strategies to target or normalize D3 receptor function in L-DOPA-induced dyskinesia (LID), where ↓ indicates reduction; ↑ indicates increase; - indicates no effect on LID; = indicates no change in L-DOPA efficacy; ? indicates not reported. MFB (medial forebrain bundle); 6-OHDA (6-hydroxydopamine); MPTP (1-methyl-4-phenyl-1,2,3,6-tetrahydropyridine).

**Table 2:** Non-pharmacologic strategies to target D3R.

Strategy	Model	Effect on LID	L-DOPA Efficacy	Ref.
Global knockout	Striatal 6-OHDA D3R -/- mice	↓ development	=	[48]
Striatal knockdown	MFB 6-OHDA rats	↓ development	=	[55]
Cell-specific striatal knockdown	MFB 6-OHDA D1R-Cre rats	↓ development	=	[58]

Summary of non-pharmacologic strategies to target or normalize D3 receptor function in L-DOPA-induced dyskinesia (LID), where ↓ indicates reduction; ↑ indicates increase; - indicates no effect on LID; = indicates no change in L-DOPA efficacy; ? indicates not reported. MFB (medial forebrain bundle); 6-OHDA (6-hydroxydopamine); MPTP (1-methyl-4-phenyl-1,2,3,6-tetrahydropyridine).
